# Connecting With Your Dentist on Facebook: Patients’ and Dentists’ Attitudes Towards Social Media Usage in Dentistry

**DOI:** 10.2196/10109

**Published:** 2018-06-29

**Authors:** Nilesh Parmar, Lin Dong, Andreas Benedikt Eisingerich

**Affiliations:** ^1^ Nilesh Parmar Dentistry London United Kingdom; ^2^ Imperial College Business School Imperial College London London United Kingdom

**Keywords:** social media, medical communication, dental practices, dental anxiety, Facebook, Twitter

## Abstract

**Background:**

Social media has begun to proliferate across medical areas and transformed how medical professionals serve and interact with their patients. It offers a new communication avenue that has the potential to engage patients and, hence, may be used to create value for both medical professionals and patients. In dentistry, even though patients and dentists frequently use social media in their personal lives, little is known about their attitudes and expectations toward using social media for professional interactions.

**Objective:**

In this paper, we focus on the role of social media in dentistry. Specifically, we explore patients’ and dentists’ attitudes toward social media usage and their current online behaviors in this context. Furthermore, we examine potential challenges and opportunities regarding dentists’ adoption of social media practices.

**Methods:**

This research employed a large-scale online survey of 588 patients and 532 dental professionals. We assessed the attitudes, expectations, and social media behaviors from both patients’ and dentists’ perspectives.

**Results:**

We found that more than 55% (290/532) of dentists in our sample have accounts for their dental practice on various social media platforms. Interestingly, while 73% (374/511) of patients did not expect their dental practice to have a social media presence, and 44% (207/468) thought that establishing a friendship with their dentists is not appropriate, the findings show that 36% (164/460) of patients had searched for their dentists, and 44% (207/470) of them were happy to establish contacts with dentists on social media. Furthermore, the findings highlight that patients were interested in exploring additional information such as online reviews and the qualifications of their dentists on Facebook pages. For dentists, more than half (375/432, 83%) of them in our sample thought that social media marketing is more efficient compared to traditional marketing.

**Conclusions:**

Our findings revealed some key challenges and opportunities to utilize social media in dentistry. For both patients and dentists, the role of social media in dental services remains vague, and both parties still share concerns about connecting with each other on social media platforms. However, there also exists a sizeable number of patients who are already comfortable to connect with their dentists on social media sites such as Facebook. The current findings show that there is an opportunity for dental practices to trade upon a more active social media presence for enhanced patient interaction and engagement.

## Introduction

### Background

The rise of social media has transformed how medical professionals interact with their patients and deliver different types of health care services [1,2,3]. Dentistry has been no exception [4]. The UK dental market was valued at £5.8bn and rising [5]. As with numerous other health profession contexts, social media has been noted to play an increasingly important role in dentistry [4]. This may create various challenges because, from a professional point of view, dentists must uphold the established image, principles, and procedures [6].

However, some of the critical traits of social media, such as self-exposure and self-disclosure, make established norms inadequate or outdated to help navigate daily interactions between dentists and their patients [7]. To this day, we still lack a clear understanding of how dentists and patients think of social media usage in dentistry. To what extent do patients and dentists feel comfortable to connect with one another on social sites such as Facebook? This research explores the attitudes of dentists and patients toward social media as well as their current online behaviors to uncover key challenges and potential opportunities for using social media in this professional context.

On social media, individuals construct public or semipublic profiles that enable them to create, circulate, share, and exchange information with their connections [8,9]. In dentistry, social media has been used in training and development of dental professionals for some time [10]. Dental education, for example, has relied on online communication to help professionals to develop clinical skills [11]. Previous studies have also highlighted that social media assists dentists to share domain knowledge with each other and facilitate professional networks [12]. It allows professionals from different locations to connect with one another and to discuss important issues and obtain feedback. These activities benefit lifelong learning and professional development [13]. That said, while social media has been hailed for effective peer-to-peer exchange, the role of social media for dentist-patient exchanges has been less than clear.

Interestingly, even though the literature has advocated the use of social media for interactions between medical professionals and patients, so far, professionals have been somewhat conservative and reluctant to involve social media in their workplace [10]. This is perhaps not too surprising. Medical services are associated with professional, formal practices while social media is usually depicted as an informal and entertaining platform [14]. Besides, personal information and communication on social media are visible to the public [15]. As a result, inappropriate social media practices might threaten medical professionals’ identities and image [15,16]. This might also create concerns for patients about privacy issues such as whether dentists use their information on social media [17]. Surprisingly, to this day, little research exists on patients’ and dentists’ attitudes toward social media usage in the context of dentistry.

Lack of insights into the attitudes of dentists and patients toward using social media in dentistry may prevent dentists from taking advantage of social media activities that might benefit both dentists and patients. For instance, social media offers a space to communicate and reach out. It empowers both dentists and patients to connect with each other without much time and distance limitations [9].

As an effective and relatively inexpensive means of communication, dental professionals can use social media for marketing activities [2]. Traditionally, dental practices would primarily advertise their services using local yellow pages (ie, telephone directory of local businesses and services) or put the promotion information on a small note in the window of dental surgeries. In the recent twenty years of digital transformation, social media has offered a forum for public communication that dentists can leverage to conduct marketing activities in a timely and cost-efficient way [4,16].

Furthermore, the communication space provided by social media allows dentists to diversify the traditional dental services by, for example, disseminating dental health information or offering online consultation [12,13,17]. Importantly, dentists can play a more active and significant role to cope with some enduring challenges in dentistry such as dental anxiety, a strong negative feeling toward visiting dental surgery that prevents patients to anticipate proper treatments [16].

Acknowledging the importance of social media in marketing and service delivery in dentistry, however, we still lack a clear understanding of patients’ and dentists’ attitudes toward social media usage [4]. Broad and accelerated access to information creates challenges for dental professionals to manage professional image and their relationship with patients [10,18]. Even though the governing institutions have realized this challenge, the published guidelines on how medical professionals interact with patients on social media are broad and, thus, may not provide feasible solutions [19].

Without a grounded understanding of dentists’ and patients’ attitudes and current usage behaviors, it is difficult to develop viable guidance that can help dentists to explore and trade upon the potential opportunities that social media platforms offer. We lack extensive knowledge about social media usage among patients and dentists. Therefore, the current research aimed to complement and extend existing knowledge and to address some of these critical issues.

### Objective

The objectives of this study were to address the following two critical points:

1. To explore patients’ and dentists’ attitudes toward social media usage in dentistry.
2. To discover potential opportunities and challenges for dentists to adopt social media practices.

## Methods

As part of this research, we conducted a large-scale online survey of patients and dentists. The dentists we target were from a general dental council or the equivalent registration. There were no specific criteria for selecting the patients. We designed separate surveys for patients and dentists with similar themes. The principal goal was to compare the responses of patients and dentists regarding their attitudes and behaviors related to the social media usage in dentistry.

The dentist survey consisted of 27 questions, with the estimated time to complete the survey to be around 15 minutes. The patient survey had 20 questions with some being split into sub-questions. The estimated time to finish the survey was around 12 minutes. We used the software Qualtrics survey platform because it is accessible with no requirement for the participants to register. Furthermore, it is available on multiple platforms including smartphones, tablets and desktop computers and, thus, helped us to encourage more participation in this study.

The survey for dentists was distributed via a dentist-only Facebook group called “For Dentists, by Dentists”. This group has over 4,500 dentists as members and currently is the most active online forum for dentists in the UK. Also, the first author promoted the survey through various avenues such as professional blogs and websites such as dentistry.co.uk, where members of the “For Dentists, by Dentists” Facebook group often visit. Specifically, the survey was aimed at the dental community, which comprises all those with a General Dental Council registration. It encompasses dentists, nurses, dental technicians, and hygienists. The key aim here was to assess dentists’ participation in and attitudes about social media and to assess to what extent this participation extends to their patients.

We promoted the survey for patients mainly through social media platforms. The patient survey was distributed via Facebook, LinkedIn, and Twitter messages by the first author, with all recipients being asked to share the survey with their connections. Furthermore, in the first author’s dental practice, the survey was uploaded onto an iPad, and patients were encouraged to fill in the survey while they waited for their appointments. The principal goal here was (1) to assess patients’ expectations of the social media presence of their chosen dental practice, (2) to determine what content on a Facebook dental practice page that patients find relevant and important, and (3) to explore patients’ view toward communicating with their dentists via social media. Both surveys ran for 6 weeks with regular updates on social media at premeditated times to enhance visibility within individuals’ timelines and increase effective response rates. This study obtained ethics approval by Imperial College London.

## Results

### Overview

**Figure 1:**
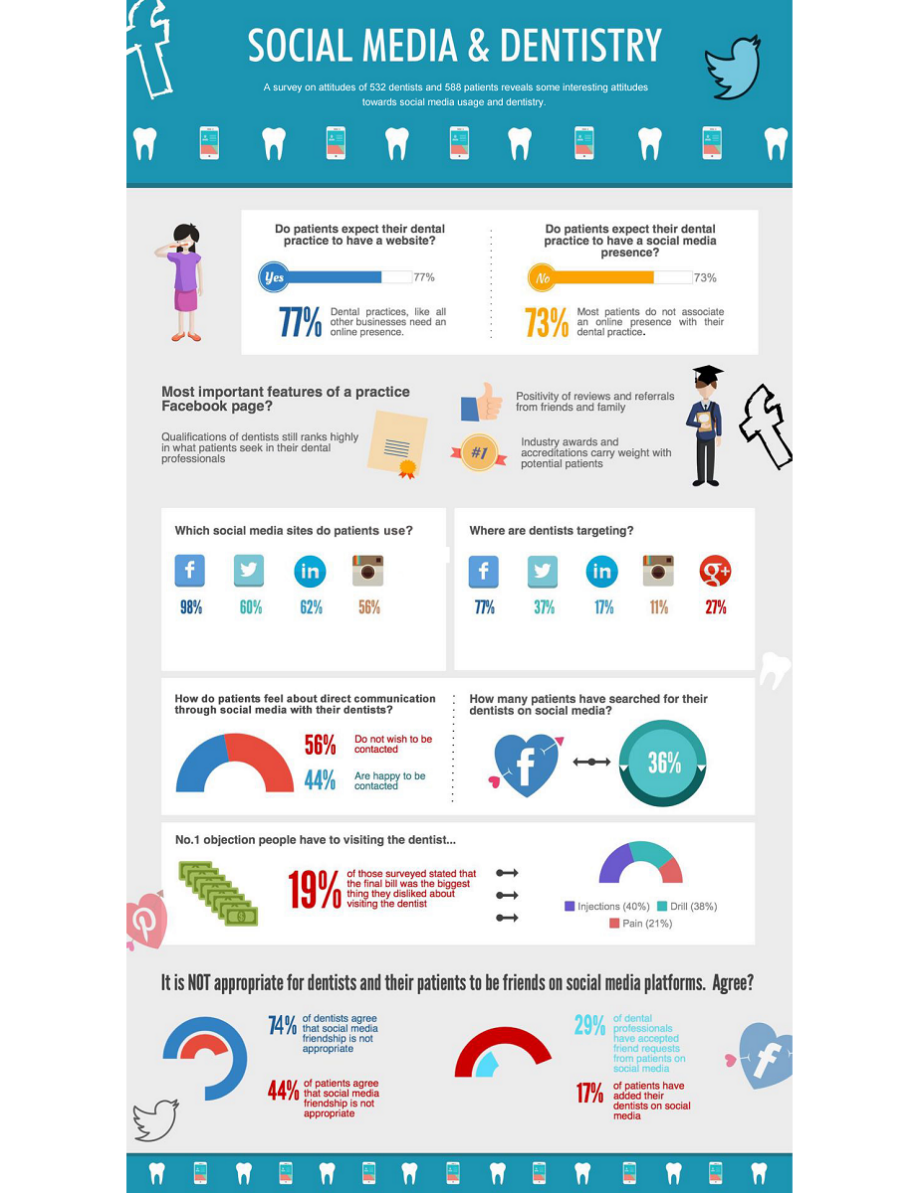
A summary of findings between dentistry and social media.

The data collection efforts resulted in 588 patients’ responses and 532 dentists’ responses. Because some respondents skipped a few questions, we highlighted the number of responses for each question when discussing the detailed results to ensure accuracy. Figure 1 summarizes some of the key insights that emerged from the data. Specifically, 77% (399/515) of patients expected their dental practice to have a website, but the majority (374/511, 73%), did not expect their dental practice to have a social media presence. That said, 44% (207/470) of patients noted to be happy to be contacted by their dentist through social media and 36% (164/460) have searched for their dentist on social media. Interestingly, while 74% (333/448) of dentists and 44% (207/468) of patients state that it is not appropriate for dentists and their patients to be friends on social media platforms, 29% (112/382) of dentists and 17% (76/460) of patients have accepted friend requests have added their dentist on social media already. We elaborate on additional vital insights in the following section.

### Platform Usage by Both Patients and Dentists

Facebook was the most popular platform to use for both patients (482/492, 98%), and dentists (290/377, 77%). For patients, Twitter, Instagram, and LinkedIn were popular choices in their personal lives. For dentists, except for Facebook, it is noteworthy that 37% (138/377) of them opened a business account on Twitter and 27% (103/377) on Google+ (see Figure 1). In general, we had a balanced response regarding the gender of both patients (321/572, 56% were female) and dentists (245/532, 46% were female) in our survey. Table 1 shows the demographic and summary statistics of survey respondents. Most of the responses were over 25 years old and owned at least one personal social media account.

### Patients’ Attitudes Toward Social Media Usage in Dentistry

**Figure 2:**
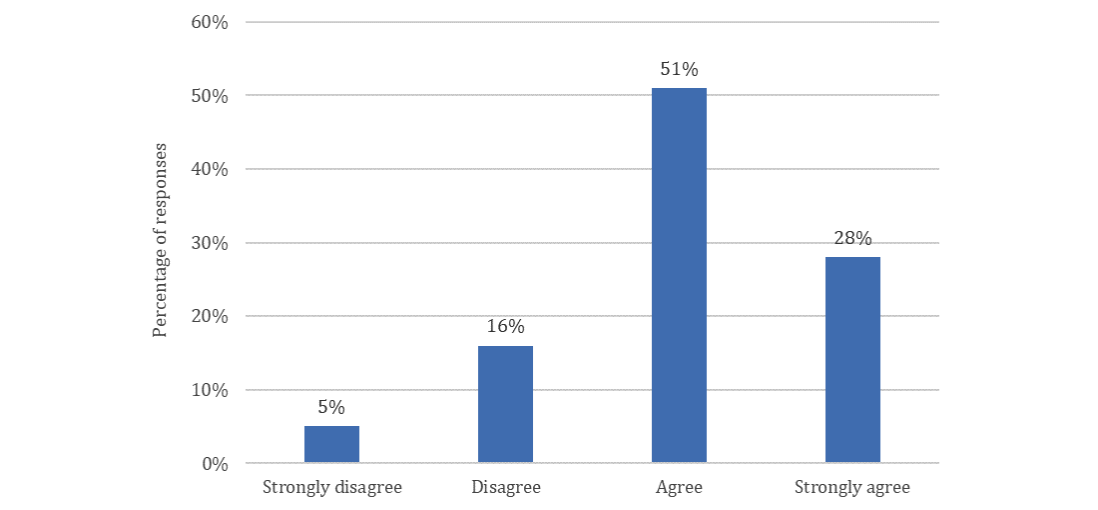
Responses from patient survey prompt: A modern-day dental practice should have an online presence (N=493).

The findings show that 47% (238/508) of patients have visited their dental surgery’s Facebook page or website. A total of 17% (76/460) searched their medical doctors or dentists on social media and added them as friends. Of patients, 19% (88/460) searched for their medical doctors and dentists on social media but did not add them as friends on social media. A total of 64% (296/460) neither searched nor added their medical doctors and dentists as friends on social media (see Table 1). Furthermore, the findings reveal that 79% (391/493) of the patients agreed dental surgeries should have an online presence of sort (see Figure 2).

**Figure 3:**
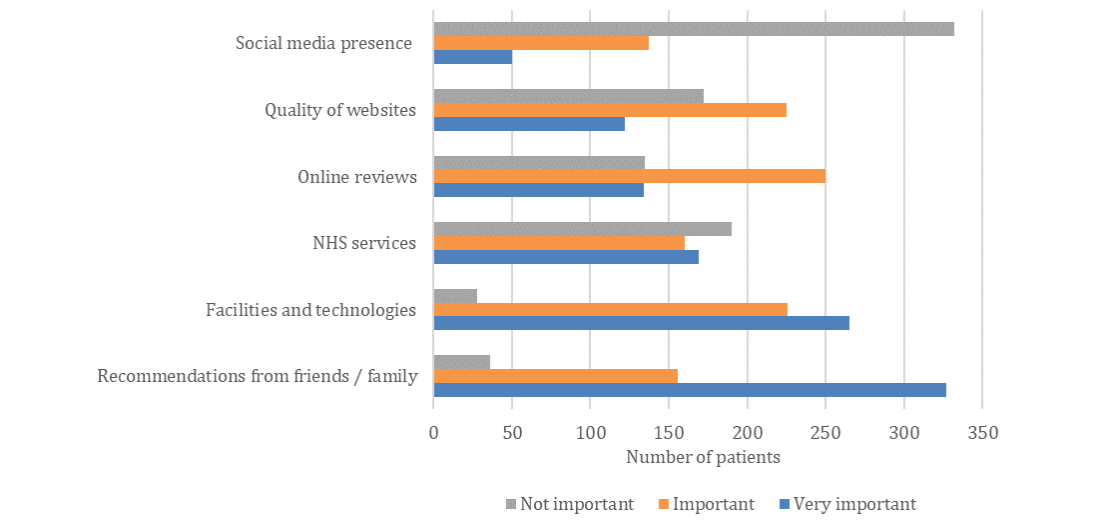
The results of a patient survey. Factors that are most important to patients when choosing a dental practice.

Moreover, for patients, our results show that social media does not play a significant role in their decision-making process when selecting a dental practice (see Figure 3). More specifically, patients valued recommendations from friends and family, facilities and technologies, online reviews and quality of websites more than they did a dental practice’s social media presence.

**Figure 4:**
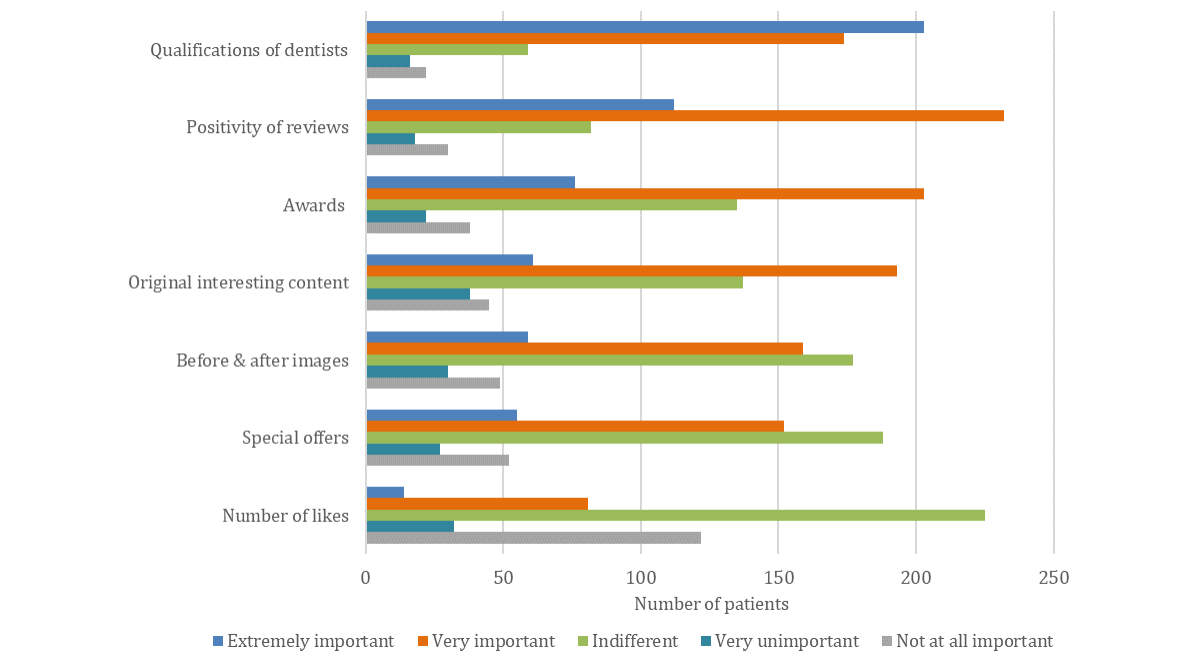
Results of a patient survey. Factors that are most important to patients when looking at a Facebook page for a dental practice.

Regarding dentists’ Facebook page, patients ranked qualifications as the most important content to be displayed. Besides qualifications, for some patients, positive reviews, awards, and original content were also appreciated on dentists’ Facebook pages (see Figure 4).

**Table 1:** Patient survey and dentist survey: demographics and personal social media usage.

Survey Variables			Dentist Survey, n (%)	Patient Survey, n (%)
**Demographic**				
	**Gender**		N=532	N=572
		Male	287 (54%)	251 (44%)
		Female	245 (46%)	321 (56%)
	**Age group**		N=529	N=588
		18-25	9 (2%)	65 (11%)
		26-35	331 (62%)	234 (40%)
		36-45	130 (25%)	172 (29%)
		46+	59 (11%)	117 (20%)
**Social media**				
	**Do you have** **a** **personal** **social media account?**		N=451	N=499
		Yes	421 (93%)	484 (97%)
		No	30 (7%)	15 (3%)
**Patients’ behaviors on social media**				
	**Have you** **visited** **your** **dental** **surgery’s** **Facebook** **page** **or** **website?**		N/A^a^	N=508
		Yes	N/A	238 (47%)
		No	N/A	270 (53%)
	**Have you ever added any of your medical doctors or dentists on social media?**		N/A	N=460
		Yes, searched and added	N/A	76 (17%)
		Yes, but only searched	N/A	88 (19%)
		Neither	N/A	296 (64%)

^a^N/A: not applicable.

An interesting finding is that when asked about the objection to visiting a dental practice, patients showed greater pain-related anxiety caused by, for example, injection and drill, compared with financial-related anxiety (see Figure 1). There seems to be an opportunity for dentists to help alleviate patients’ concerns and anxieties. Social media might offer a useful tool in helping patients access critical and helpful information about dental solutions and access these in a convenient and non-threating way. Next, we explore the insights we obtained from our survey with dentists.

### Dentists’ Attitudes Toward Social Media as a Marketing Tool

**Figure 5:**
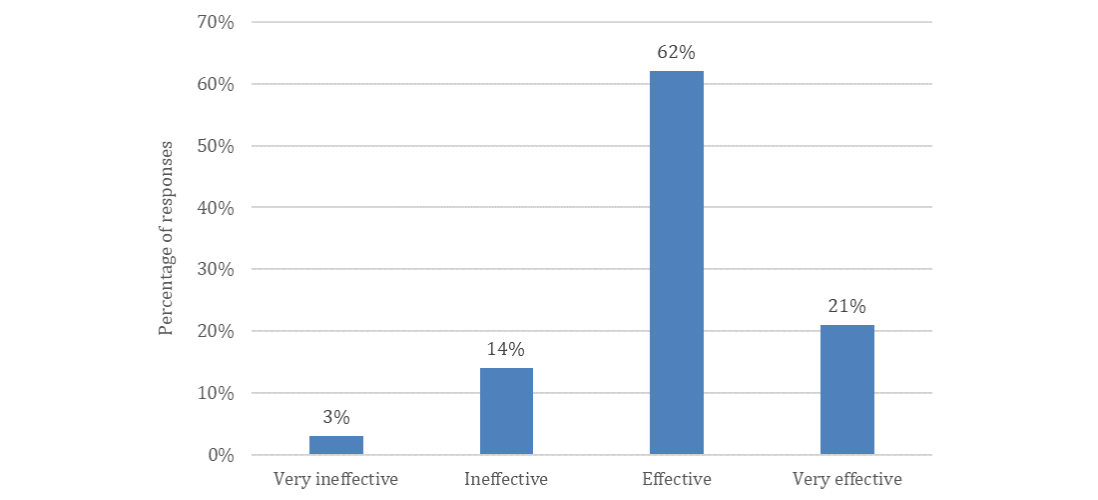
Responses from patient survey prompt: How effective is a social media presence for a dental practice to engage and obtain new patients (N=432)?

**Figure 6:**
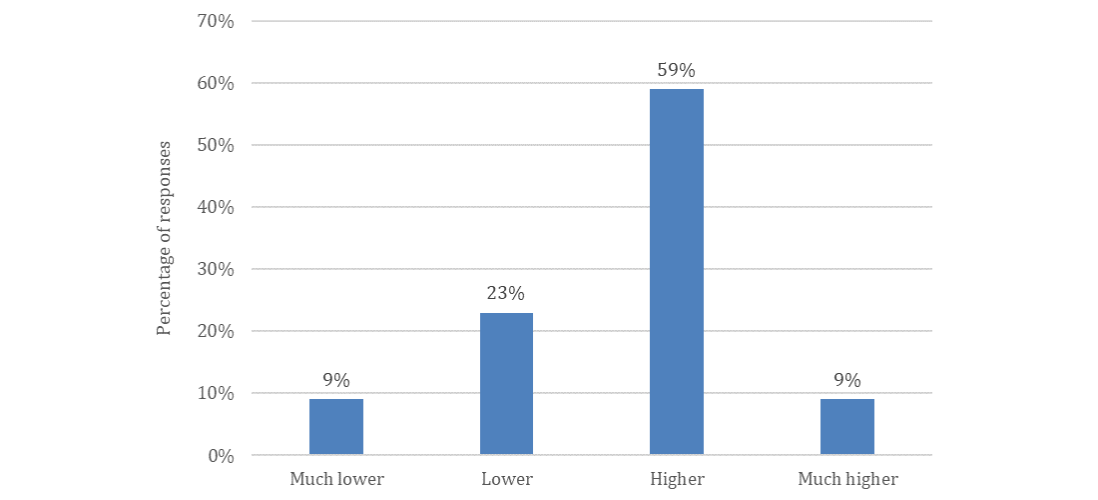
Responses from patient survey prompt: What do you think is the return-on-investment of social media marketing when compared to conventional marketing for a dental practice (N=422)?

The findings reveal that dentists hold a positive attitude toward social media as an effective tool to reach new patients (see Figure 5). Critically, the return on investment (ROI) of social media marketing was noted as higher than for traditional marketing efforts. Hence, potentially, social media can improve the financial performance of dental practices (see Figure 6).

## Discussion

### Principal Findings

The use of social media as a means to facilitate communication between dentists and their patients has been somewhat limited [10]. Social media, however, may offer critical opportunities for dentists to facilitate patient-dentist relationship outside the surgery [20]. That said, we did not know much about how patients and dentists feel about direct communication with each other on social media. Before this study, we conducted a PubMed search, which resulted in a total of 179 articles with the search term of “social media and dentistry” and the conditions of “published in the recent ten years” and “full text available”. Only a few articles discuss the role of social media in dentistry, and the empirical samples of these studies are relatively small. The present research thus complements and extends the extant body of work by surveying the attitudes and preferences of 588 patients and 532 dentists. It reveals interesting and some surprising insights into attitudes toward social media and dentistry.

In line with prior work, our results show that both patients and dentists are already active on various social media platforms to some extent [4,15]. However, they share some concerns about connecting with each other as online friends. Although dental professionals see social media as an essential opportunity to improve marketing efficiency, we find that patients have unclear expectations about how social media adds value to them. Despite these challenges, we have also uncovered many key opportunities that offer critical insights for dental professionals.

### Patients’ Relationship Status With Dentists on Facebook: It’s complicated

The current findings showcase that both dentists and patients remain somewhat hesitant to connect with each other as friends on social media. This finding is in line with previous work, which noted that the trait of self-disclosure might increase both parties’ concerns about their privacy [1,10]. Furthermore, social media, such as Facebook, is typically used to connect with friends and family [21]. Hence, relevant social media practices and routines have been developed outside the professional context—that is, what is appropriate to be shared on social media may not necessarily be appropriate in a more professional context [9]. That said, a surprisingly high number of patients already connect with dentists on Facebook. For instance, 44% (207/470) of patients indicated to be happy to be contacted by their dentists on social media. Thus, avenues to explore this exciting issue further do exist.

When moving from interpersonal to professional interactions, intuitively, the relationship between patient and dentist often does not equal to “friendship”. Thus, the routines on social media such as commenting on and liking each other’s posts may not be easily applied in a professional context. It may confuse patients and dentists about the type of relationships they should hold and how to interact with each other on social media. That said, without the capability to connect with patients on social media, it would be challenging for dentists to leverage social media as an effective marketing tool. Patients perhaps also miss the opportunity to have better access to their dentists and obtain relevant and timely information about dental services that can help alleviate their anxieties and fears about their next visit to the dentist.

### Reaching Out to Patients Via Social Media

The findings highlight that dentists value social media as useful marketing tool. On the contrary, as the target audience, most patients do not consider social media as part of their decision-making process. However, 76/460 (17%) of patients in our sample declared that they had added their dentist on social media. Thus, while some patients do not seem to see the value of a social media presence by dental practices, there also appears to be an opportunity for dentists to trade upon a more active social media presence.

Critically, patients appear not to know why they should use social media when selecting a dental practice. Such ambiguity and mismatched attitudes present a barrier for dentists to take advantage of social media fully. Dentists need to think about and explicitly suggest how social media offers exclusive services and information for patients that are unavailable on other channels (eg, website or phone call). For example, YouTube is perceived and widely acknowledged as a convenient educational content sharing platform for dentists [22]. Dentists can communicate with their patients about what information or services patients can obtain on Facebook, Twitter or other social media platforms.

### Opportunities for Dentists and Patients to Connect on Social Media

The findings indicate many opportunities for dentists and patients to connect on social media. First, dental professionals may use LinkedIn to showcase their expertise. Based on our findings, patients would like to know dental professionals’ qualifications before they visit dental practices. As one of the popular social media platforms that patients use, LinkedIn operates the world’s largest professional network [23]. It allows dentists to display their expertise including educational background, work experience, skills, and endorsements. The norms and codes of this particular platform are consistent with the professional image of dentists [24]. It also minimizes dentists’ concerns about invasions of their personal lives. Thus, LinkedIn naturally serves as a professional space that dentists can use to demonstrate their expertise and capabilities, as part of social media marketing activities.

Second, dentists may connect with patients on social media to encourage online word of mouth. As the most critical factor that impacts patients’ decision-making, recommendations from friends and family could be operated on social media platforms. Compared to offline word of mouth, recommending dental practices on social media allows patients to share more information with their peers when necessary. For example, a patient may send the website of a recommended dental practice to her peers directly and encourage them to obtain more information by visiting the website. Social media also allows various forms of word of mouth such as reviewing dental practice’s service and reposting its advertisements. Also, activities such as commenting on the posts and simply “liking” the posts increase a surgery’s awareness within its patients’ social network [21].

Third, since patients are aware of and visit the websites of their dental practices, dentists might introduce their social media account on the website. They should state the functions of each social media platform explicitly. For example, some banks guide their customers to use Twitter for online help by adding the link to their Twitter page under the tab of “Help” [25]. That is, customers see the bank’s Twitter page as a functional place to solve their questions. By introducing the functions of social media platform, patients would have a clear idea of how social media creates value for them (eg, a place for online Q&A or a place for finding helpful or encouraging daily dental health suggestions). Also, integrating social media platforms with a website also increases the chance for patients to be aware of the social media presence of their dental practice.

### Limitations and Future Research

We note the following limitations of our study, which also offer promising avenues for future work. First, in the data collection process, asking some of the respondents to use a tablet to fill in the questionnaires might introduce a particular bias. For example, respondents may not be used to these devices and skip questions. That said, the answers from respondents who filled out the survey on a tablet did not differ from respondents who filled out the survey through other means in our study. Second, we did not collect qualitative data. For example, 74% (333/448) of the dentists agreed that connecting as friends on social media is inappropriate. Integrating a qualitative method such as in-depth interviews would allow us to explore dentist’s concerns in more detail.

Also, a qualitative method may allow us to analyze successful cases of social media usage in dentistry. It would provide some specific suggestions for dental professionals. Moreover, the analyses of our data suggest that social media usage in the context of dentistry and patients did not significantly differ for female versus male or older versus younger patients. This is intriguing and warrants further investigation. What other patient characteristics may influence attitudes toward social media usage in the context of dentistry? We invite additional research to address these relevant issues. Our research thus suggests several promising avenues for future studies.

Social media provides opportunities to tackle some issues such as after-clinic care and dental anxiety. Dental anxiety, an enduring challenge in dentistry, often causes sleeplessness, reluctance to form close interpersonal relationships and problems in workplaces [22,26,27]. It acts as a barrier for patients to actively seek necessary dental treatments [28]. Unfortunately, despite advances in technology, the prevalence of dental phobias has not been changed in the last twenty years [26]. Coping with it requires professional help, especially before clinic visits. Social media may allow dental professionals to deliver the help outside a dental practice environment. Notably, some features of social media such as gamification [29,30] and engaging website design [31] may be particularly relevant to address and inform patients about critical health-related issues and educate them, which can also help increase their trust and appreciate the level of service received [32-34]. Research studying the role of how social media may help reduce patients’ dental anxiety is a worthwhile and exciting endeavor.

Furthermore, future research may also consider the role of professional and regulatory bodies. Social media usage might threaten privacy and bring new challenges for both patients and dental professionals. For dental professionals, the principles, practices, and procedures related to social media communication are not clear [15]. For example, dentists may use social media to demonstrate their expertise by uploading clinical photographs of before and after treatment. Is it acceptable? If so, when? Moreover, unprofessional audiences may challenge dentists’ expertise and capabilities in a public place [15,35]. These potential violations need to be considered carefully and managed by professional and regulatory bodies to ensure that social media is a safe place for dental professionals in which to engage.

Currently, the General Dental Council (GDC), the regulatory body for dentistry in the UK, has published a guideline in 2013 titled “Guidance on Using Social Media”. It takes a hardline approach to its registrants [19]. For example, the document states: “maintain appropriate boundaries in the relationships you have with patients” and “you should think carefully before accepting friend requests from patients” [19]. The British Medical Association (BMA) also suggests that accepting friendship requests from patients might be inappropriate [36]. Further research may explore the type of guidance that professional and regulatory bodies should provide. That is, they should navigate dentists’ daily social media activities that not only ensure the alignment with ethical codes but also leave sufficient room for them to use social media to create value for their patients [36].

Future research may explore some of the critical skills and mindset that young professionals need to flourish in the digital era. Medical and pharmaceutical students are not always clear about the long-term repercussions associated with their online behaviors [37]. Merely listing acceptable social media practices may not work, as unfavorable outcomes can be caused for various reasons [38]. For example, users can upload photographs and thoughts on social media within seconds, without evaluating whether the message aligns with their professional image. These messages are also visible electronic footprints that might have unintended and negative consequences [15]. Social media usage may also create an environment where patients feel more comfortable to participate and proactively ask questions [39,40] or even challenge others [41]. Thus, while social media can facilitate transparency and allow people to access information conveniently [42,43], which allows service providers to be seen as more helpful and hence play a more critical role in people’s lives [44-46], true engagement online and offline remains a challenge [47,48]. Future research that studies how the current medical education may better help professionals and equip them with the skills needed to reach out to patients and manage the relationships with their patients in the context of social media is richly deserving.

Also, advances in technologies such as artificial intelligence might impact patients’ trust and behaviors significantly or even shift the extant business models for high credence services such as dentistry. Given the importance of educating one’s customer [49,50], it is critical to start to think about how young professionals can develop a technology-sensitive mindset as a long-term professional asset from their professional education and training to share critical information with and earn stronger trust form their patients. Such technology-sensitive mindset can help medical professionals navigate a hard-to-predict environment [51,52] and engage in responsible behavior while trading upon social media offerings (eg, visually pleasing profile with enticing logo) to explore new opportunities to connect with their patients more effectively [53-55]. As current research shows, people can become instantaneously attached to, and find it hard to give up, the digital services they use [56]. Future work that explores how dentists can make use of social media and other mHealth solutions to further their self-identification with their profession and pride [57] and ensure patients lead healthier lives [58] is richly deserving.

### Conclusion

A total of 207/470 (44%) of patients noted to be happy to be contacted on social media by their dentists, while 164/460 (36%) of patients have already searched for their dentist on social media. Social media offers opportunities for dental professionals to improve the efficiency of marketing activities, and to provide additional services such as offering online diagnosis and online Q&A. However, within the professional context, the norms and procedures related to social media usage in patient-dentist communication remain vague and underdeveloped. Moreover, while 74% (333/448) of dentists agreed that social media friendship was not appropriate, 112/382 (29%) accepted friend requests from their patients on social media. Our study represents an important step to unpack patient and dentist expectations about, and attitudes toward, social media usage in dentistry. We hope additional work can build on some of the current findings and shed additional light on the role of social media in dentist-patient interactions and relationships.

## Abbreviations

BMA: British Medical Association
GDC: General Dental Council
ROI: return on investment
